# Climate variability links to changes in Rioja wine (Spain)

**DOI:** 10.1007/s00484-024-02816-0

**Published:** 2024-11-15

**Authors:** Domingo F. Rasilla, Raquel Aransay, Francisco Conde-Oria

**Affiliations:** 1 Departamento de Geografía, Urbanismo y Ordenación del Territorio, Universidad de Cantabria, Santander, 39005 Spain; 2 Departamento de Transporte Público, iPlan Movilidad, Madrid, 28045 Spain

**Keywords:** Rioja wine, Climate variability, Leaf area index (LAI), Low-frequency variability

## Abstract

This study investigates the impact of regional climate changes on the production, quality, chemical composition, and phenological patterns of Rioja wine in Spain from 1993 to 2017. Data from DOCa Rioja and the Marqués de Riscal winery were analyzed in conjunction with meteorological and remote sensing data to provide a comprehensive evaluation. The findings reveal an increase in alcohol content and pH, coupled with a decrease in acidity, correlative to phenological shifts such as earlier grape ripening and reduced leaf canopy. Additionally, a thorough examination of monthly climate anomalies highlights the significance of May in determining harvest outcomes, influenced by the Eastern Atlantic (EA) mode of low-frequency variability. The potential connection between springtime weather conditions and tropical climate variability is also explored.

## Introduction

Viticulture is a highly profitable agricultural sector globally, playing a crucial role in the economies of many wine-producing regions through exports and job creation. These regions also boast cultural landscapes shaped by viticultural practices (Lourenço-Gomes et al., 2015). As a sector sensitive to climate conditions, viticulture serves as a valuable indicator of both historical and current climate fluctuations (Chuine et al., 2004). Extensive research has explored the impact of weather on wine yield (Lorenzo et al., 2013), quality (Davis et al., 2019), composition (Teslic et al., 2018) and phenology (Ramos et al., 2018) highlighting the potential threats posed by global warming to established wine-producing areas. Projected climate changes not only present economic risks but also jeopardize the unique landscapes where grape varieties and their origins are central to the value and identity of wine (Schamel and Anderson 2003).

Given the economic significance and climate sensitivity of the wine industry, it has been at the forefront of developing innovative agricultural tools and practices within the framework of precision agriculture. These advancements aim to enhance productivity while minimizing environmental impacts and reducing reliance on pesticides and fertilizers. Real-time phenological data obtained through remote sensing have become crucial for achieving these goals (Mulla 2013). The Normalized Difference Vegetation Index (NDVI) is a widely used remote sensing-based biophysical index that reflects vegetation health and greenness. Another important index is the Leaf Area Index (LAI), which measures the leaf area per unit of ground surface area. Remote sensing-based LAI estimates provide a non-invasive and efficient means of assessing grapevine vigor, yield, and grape quality (Sun et al., 2017).

This study examines the impact of recent climate variations on the key characteristics of Rioja wines in the La Rioja region. The paper is structured as follows: Sect. 2 provides an overview of the data sources and methodologies used, Sect. 3 presents the results, and the concluding section offers a discussion and conclusions.

## Materials and methods

### Study region

**Fig. 1:**
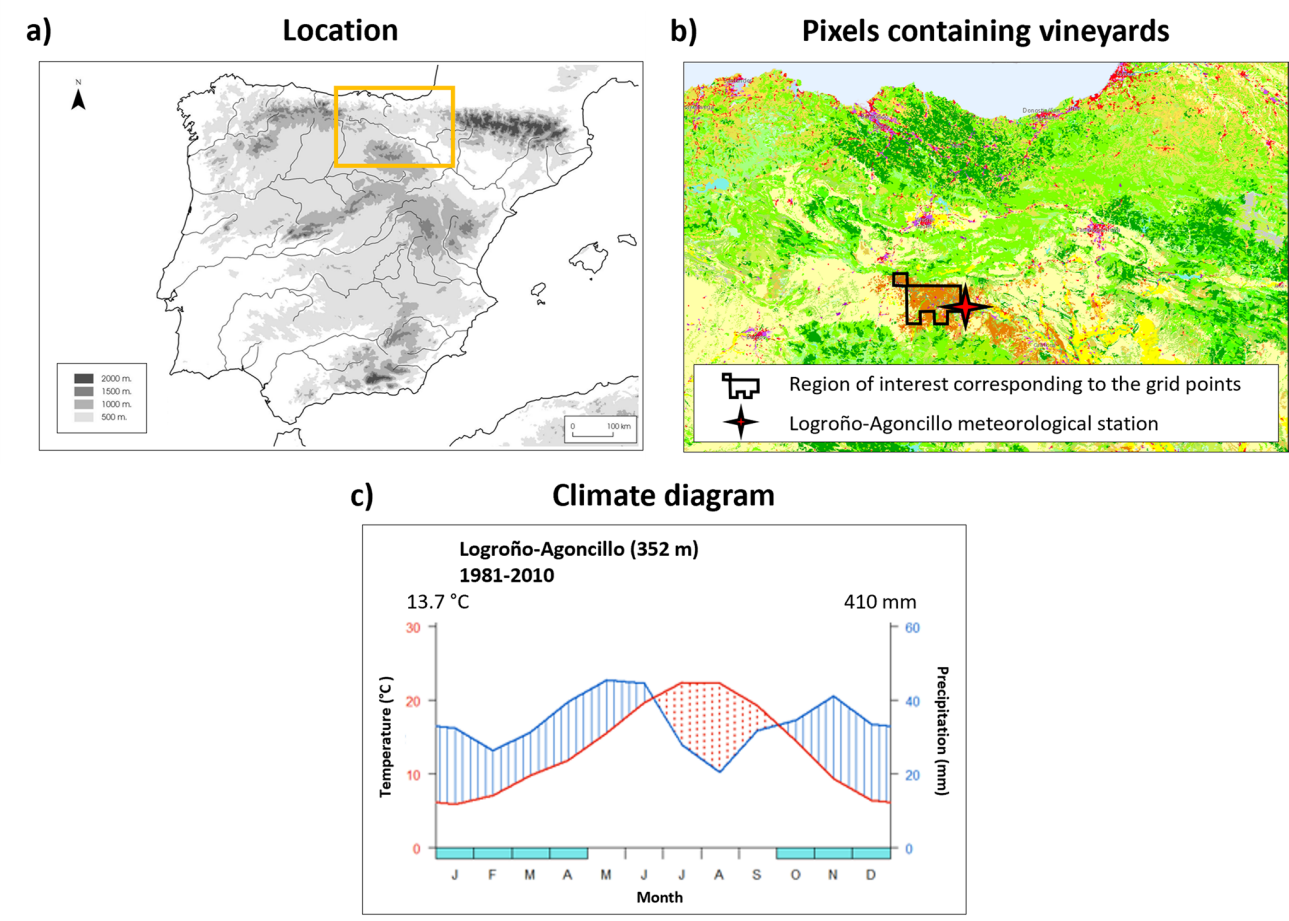
(**a**) Location of the study area, (**b**) Pixels containing more than 75% of the land cover classified as vineyards and location of the meteorological station and (**c**) Logroño-Agoncillo climate diagram

La Rioja region, a prestigious Qualified Designation of Origin (DOCa), boasts a storied past as a prominent wine-producing area in Spain. Vineyards occupy 65,000 hectares (Fig. 1), located on the glacis and river terraces of the Ebro River, reaching elevations of up to 700 m. Red grape varieties reign supreme in the region, representing 93.68% of total production, with Tempranillo and Garnacha as the primary cultivars. On average, Rioja yields approximately 250 million liters of wine annually, with roughly one-third of this volume being exported worldwide. This export quantity accounts for approximately 30% of the region’s total export value (OIVE 2024). The region features a Mediterranean climate with characteristics akin to a continental climate, marked by notable annual and daily temperature fluctuations, coupled with reduced annual precipitation (405 mm), primarily concentrated during the spring and autumn seasons (AEMet 2024).

### Wine data

The DOCa Rioja Board supplied data on vineyard area, wine production, and vintage quality spanning the years 1993 to 2017. Furthermore, grape compound information was gathered from experimental plots (bush-trained under rainfed conditions) where grape maturation is meticulously monitored. These compounds encompassed pH levels, total tartaric acidity (TTA), probable volumetric alcohol content (PVAD), and the average weight in grams of 100 grapes samples (WB100). Only data from the period immediately preceding the harvest (the latest sample collected in September) were considered in the analysis, with individual plot values being regionally averaged. Plots not cultivated with Tempranillo and Garnacha grape varieties were excluded from the study. The Marqués de Riscal winery contributed veraison and harvest dates from its vineyards for inclusion in the analysis.

### Climate data

The meteorological data used in this study were collected from the Logroño-Agoncillo meteorological station (AEMet; WMO code 08084). Daily values were averaged to obtain monthly data, which were then used to calculate various bioclimatic indices. For the growing season (April–October), the study considered the following variables: maximum (TX) and minimum temperature (TN), sunshine hours (SS), average cloud cover (CC), and relative humidity (HR). Additionally, the study calculated total hot days (HD; Tx > 30 °C), frost days (FD; Tn < 0ºC), accumulated precipitation (PP), and the number of wet days (WD; PP > 1 mm). Key indices such as the Cool Night Index (CI; Tonietto and Carbonneau 2004), Growing Degree Days index (GDD; van Leeuwen et al., 2004), Heliothermal index (HI; Huglin 1978), and Dryness Index (DI; Tonietto and Carbonneau 2004) were also considered.

Moreover, the study used Standardized Precipitation Evaporation Index (SPEI) values (Vicente-Serrano et al., 2010) from the nearest grid point to Logroño-Agoncillo to assess the impact of water deficits on vineyards (https://spei.csic.es/index.html). For analysis, the SPEI was divided into three distinct time periods: SPEI_3_ for July to September (representing summer conditions), SPEI_6_ for March to September (the growing season), and SPEI_12_ for October of the previous year to September.

Finally, the monthly indices representing the key modes of low-frequency variability in the North Atlantic were downloaded from the Climate Prediction Center (https://www.cpc.ncep.noaa.gov/data/teledoc/telecontents.shtml).

### Remote sensing data

The study utilized the GLOBMAP LAI (Version 3, Liu et al., 2021) database to examine the phenological activity of wine grapes based on LAI index values. The original data were aggregated into monthly values. The equatorial pixel’s native resolution is 0.0727273 degrees, equivalent to an area of approximately 48.3 km² at the specific latitude of interest. This resolution enables the identification of various land cover types within a single pixel. To ensure a consistent focus on vineyards, only pixels with a minimum of 75% vineyard coverage (around 42 km²) were included in the analysis. The CORINE Land Cover database (CLC; https://land.copernicus.eu/en/products/corine-land-cover) guided this purpose; vineyards are categorized as one of the 44 thematic classes in the database, with the 2018 version chosen for its accuracy.

### Methodology

A Principal Component Analysis (PCA) was conducted on phenological, wine compound, and production variables that were mean-centered. PCA is a statistical technique that condenses information from a multivariate dataset into a smaller set of new variables called principal components. These components are linear combinations of the original variables and are mutually independent. By monitoring the changes in these new variables over time, common patterns of behavior can be identified.

To compare vintages with meteorological and PCAs time series, the Kruskal-Wallis test was chosen as the appropriate statistical method, complemented by the Conover-Iman test to determine the predominant sample. Trends in the time series were detected and quantified using the nonparametric Mann-Kendall and Theil-Sen methods. A correlation analysis was carried out to investigate the relationships between the PCA time series and bioclimatic indices. To adjust for temporal trends, the partial correlation approach was utilized, with the year acting as a control variable.

However, relationships based on average or cumulative values for an entire season may mask specific associations at sub-seasonal time intervals. Therefore, an examination of the annual cycle during years associated with extreme PCA values was conducted to offer a more detailed assessment of the variables at a monthly level (Boschat et al., 2016). Composite values were computed by subtracting detrended monthly time series from the original dataset and averaging the resulting residuals across years within a specific category. The classification of each PCA score was determined by dividing the detrended time series into terciles. An extreme year was defined as one in which the PCA value exceeded or fell below the upper or lower tercile, respectively, of the time series. Confidence intervals for the composite values in both extreme categories were established by calculating the 5th and 95th percentiles from 10,000 randomly generated samples of eight years (the number of years in each tercile) using the bootstrapping method (Efron and Tibshirani 1993).

## Results

### Temporal evolution of Rioja wine characteristics

**Fig. 2:**
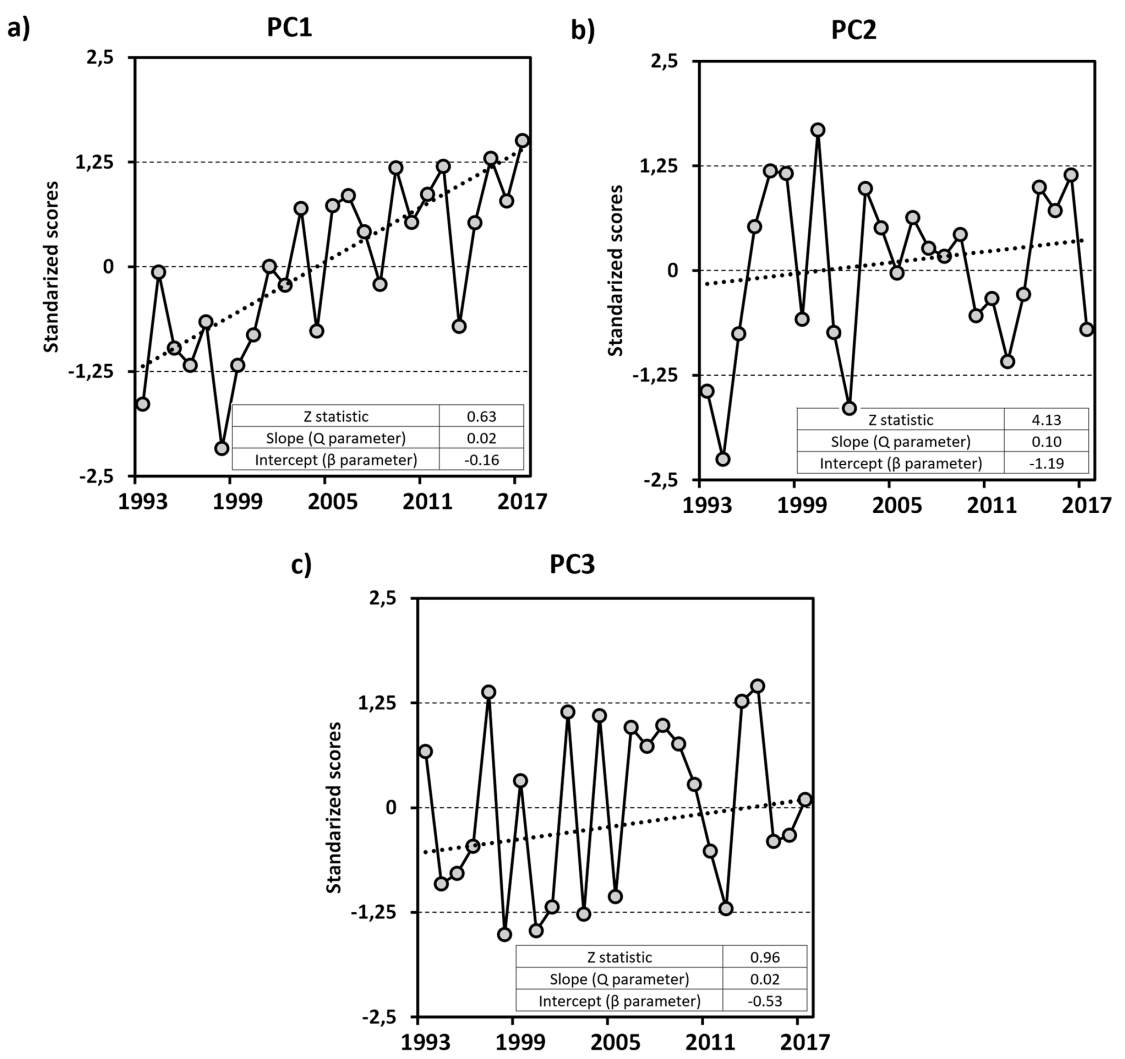
Temporal evolution of the PC scores and significance of the trends: (**a**) PC1, (**b**) PC2 and (**c**) PC3

A correlation matrix (Table 1) was analyzed using PCA, which identified three principal components (Fig. 2). Positive/negative values of the PC1 (Fig. 2a), which accounts for 49% of the total variance, characterize years with high/low alcohol content and pH, reduced/enhanced total acidity and early/late dates of veraison and harvest. The second PC, accounting for 20% of the variance, is linked to production and crop yields, while the third PC, representing 12% of the variance, is associated with berry weight. A significant increasing trend is observed only in first PC (α = 0.001 level of significance), indicating that Rioja wines are undergoing accelerated maturation, with rising pH and alcohol levels and decreasing acidity.

**Table 1:**
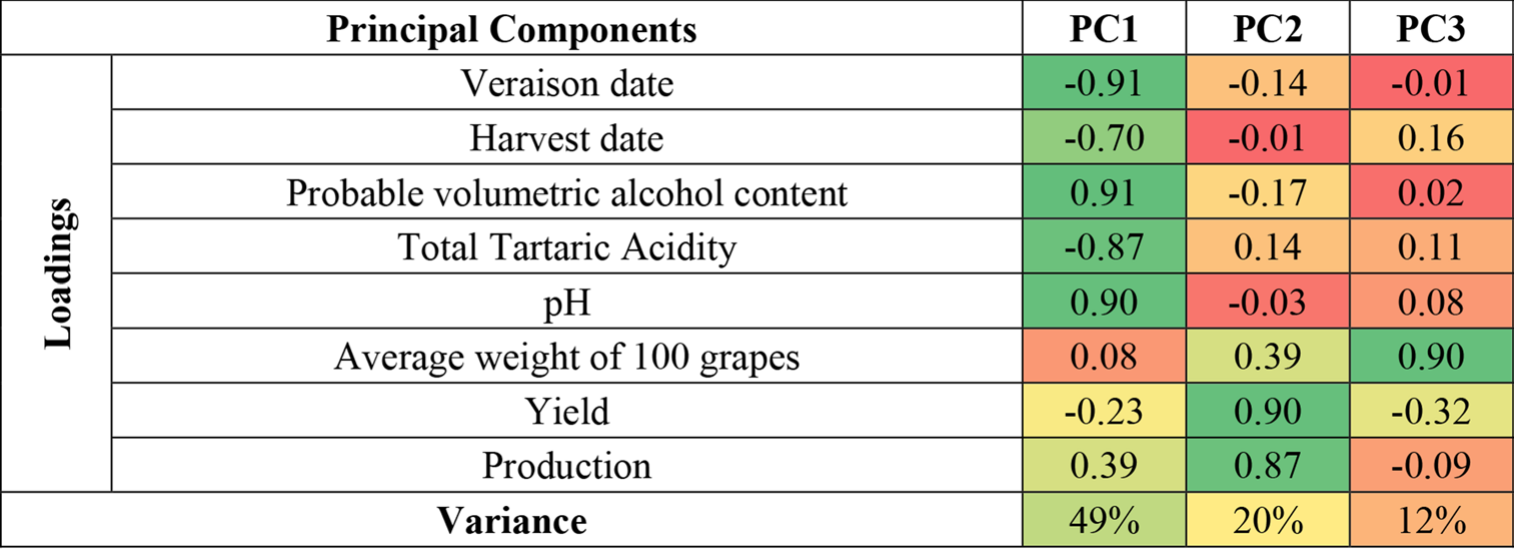
Loadings and variance of the principal components

Analysis of Rioja vintages from 1993 to 2017 shows minimal variation, with most vintages maintaining consistent quality. No vintages were rated below “good,” and seven were classified as “excellent”. The Kruskal-Wallis test did not yield statistically significant results regarding the impact of wine properties, as indicated by PC values, on vintage categorization.

### Relationships with regional climate variability

To examine the influence of climate variability on wine characteristics, each PC time series was analyzed in relation to the bioclimatic indices outlined in Sect. 2 (Table 2). As expected, PC1 showed strong positive associations with warm thermal variables, particularly maximum temperature and summer accumulated heat. The relationships with sunshine and cloud cover were less pronounced. Notably, PC1 did not exhibit significant connections with cold thermal indicators or total precipitation.

**Table 2:**
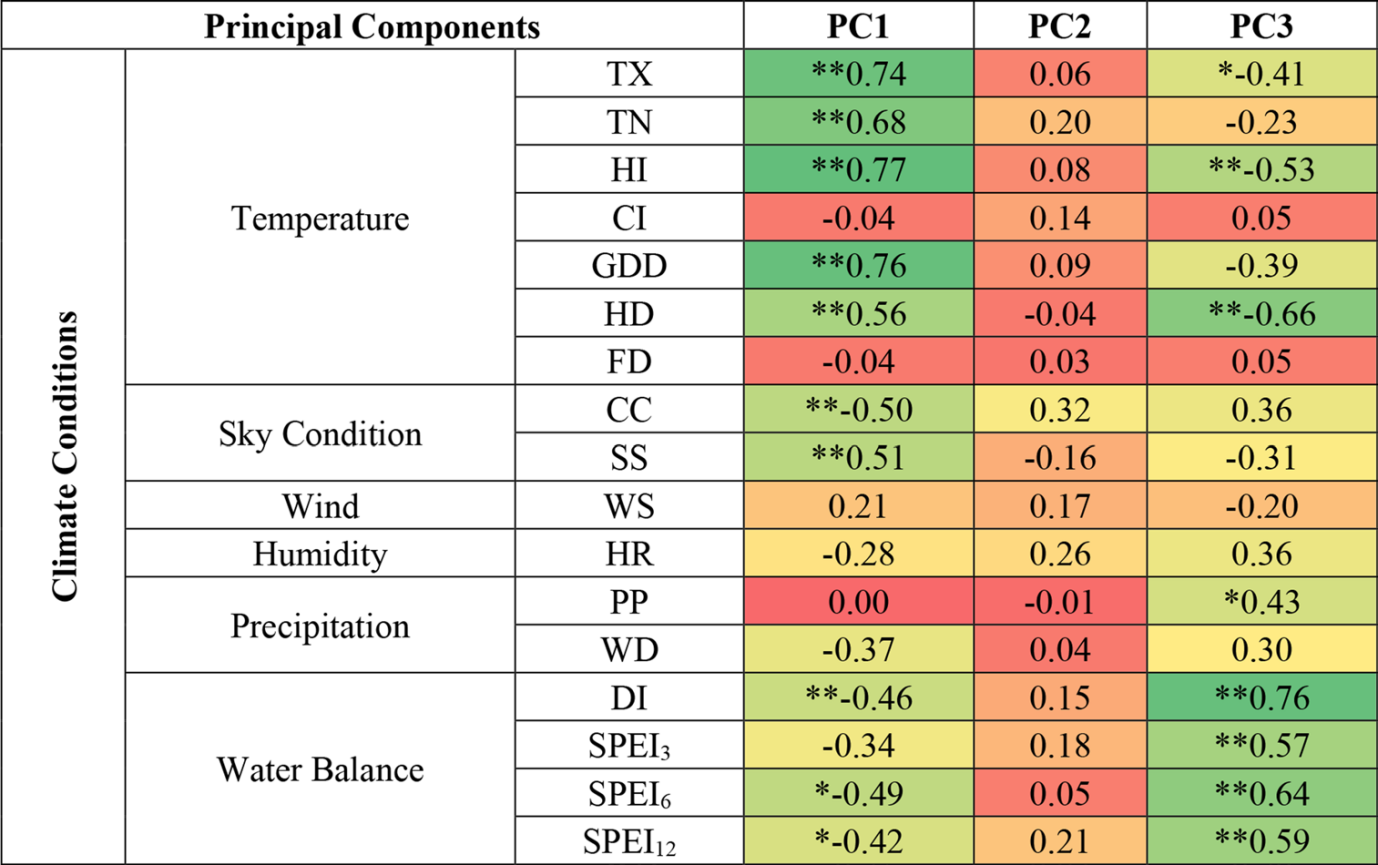
Partial correlation coefficients (with time as fixed variable) between the principal component scores time series and the corresponding climate variables time series

* stands for values statistically significant at the 95% confidence level

** stands for values statistically significant at the 99% confidence level

PC3 displayed stronger correlations with water balance variables, with the SPI_6_ (covering most of the growing season) showing the most significant correlation, as well as with other precipitation-related variables. A negative correlation was observed between PC3 and thermal factors such as maximum temperature, Heat Index, and the frequency of hot days. PC2 was excluded from further analysis due to a lack of statistically significant correlations.

Vintage categories seemed to be minimally impacted by climatic variations. The only climate variable that displayed a statistically significant difference among vintage categories was the frequency of wet days; however, this difference was significant only for the “Good” category compared to the others.

Years included within the upper tercile of PC1 were preceded by significantly warm (maximum temperatures), sunnier and dry months from May to July, while the lower tercile displayed opposite conditions only for May.

**Fig. 3:**
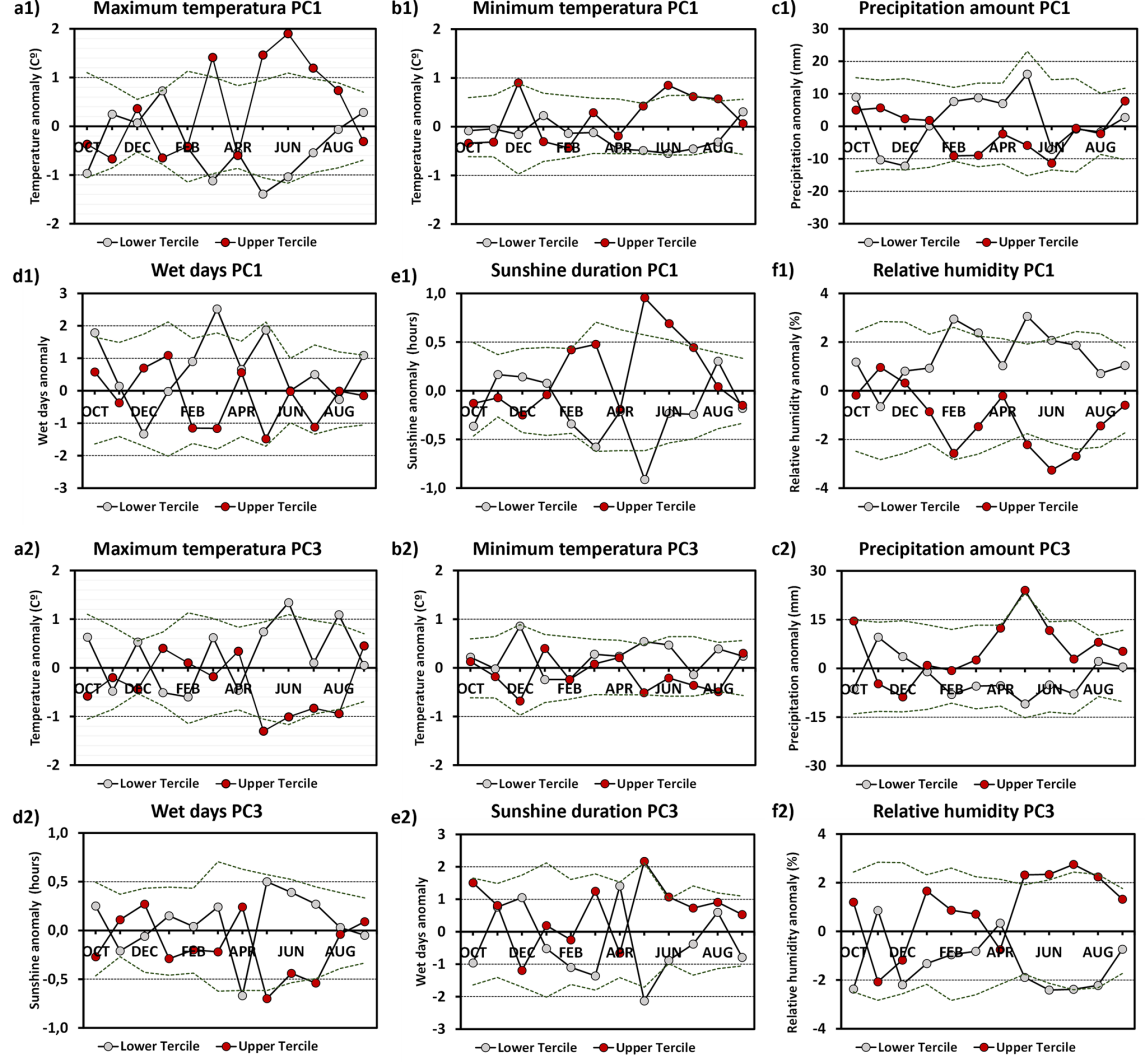
Annual cycle of monthly composites of anomalous values: (**a**) Maximum temperature, (**b**) Minimum temperature, (**c**) Precipitation amount, (**d**) Wet days, (**e**) Sunshine and (**f**) Relative humidity (%) in Logroño-Agoncillo, with respect to low and high PC1-PC3 years. Dotted lines indicate the threshold of significative anomaly values at the 5% and 95% levels of confidence, as obtained from 10, 000 random samples

Another month with an influence on PC1 is March when the budbreak stage is about to begin. Warm conditions were favorable to higher values of PC1, while cold, wet, and humid months resulted in significantly low values. Regarding PC3, the prolonged influence of relative humidity from bloom to veraison (until July) may be linked to soil water availability during a period of lower precipitation (Girona et al., 2009). The dormancy period from November to February did not exhibit significant climatic anomalies (Fig. 3).

### Wine and phenological indices

By utilizing remote sensing to derive phenological indices, a more comprehensive examination of observed trends in phenology can be conducted, enabling investigations on a larger scale. According to the PCA results, the underlying processes that are modifying the chemical composition of Rioja wine are also influencing the phenology of the vineyards.

**Fig. 4:**
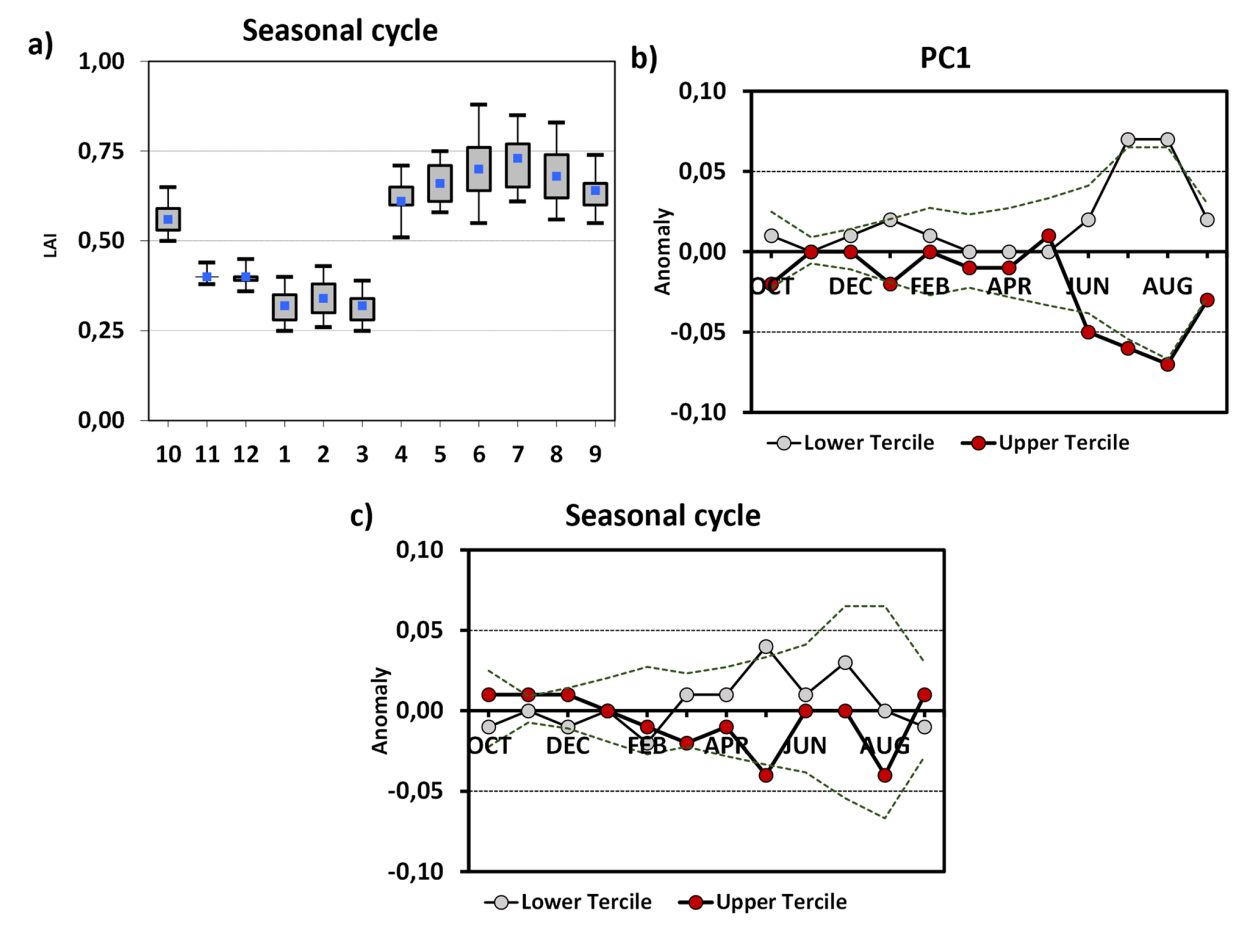
(**a**) Annual cycle of monthly LAI values and Annual cycle of monthly composites of anomalous values of LAI with respect to low and high: (**b**) PC1 and (**c**) PC3. Dotted lines indicate the threshold of significant anomaly values at the 5% and 95% levels of confidence, as obtained from 10, 000 random samples

The annual cycle of LAI monthly mean values in vineyard-dominated pixels peaks from April to October and reaches a low point from November to March, aligning with the typical vegetative cycle observed in Mediterranean climate vineyards (Fig. 4). The lowest LAI values occur during the dormant stage, starting with leaf fall and continuing through winter. The period of vigorous leaf-out and vineyard growth commences in April, followed by bloom in early June, marking the transition from vegetative to reproductive growth stages. Veraison, the development and maturation of grapes, typically occurs in early August, leading to reduced LAI values as older leaves struggle to absorb the necessary radiation for photosynthesis. Harvest, when grapes reach optimal maturity, usually takes place between late-September and October. Additionally, the plot displays fluctuations in dispersion values associated with vineyard physiological processes and regional climate variability.

Years associated with high PC1 values correspond to lower-than-normal photosynthetic activity in summer (June to August), while low PC1 values show a higher leaf canopy (July and August). No signal was found with other principal components, except for May, where elevated PC3 values coincided with decreased photosynthetic activity and vice versa. The time series of the LAI index in July and August also reveals a declining trend over the study period, reflecting the overall evolution of Rioja wine characteristics.

### Links with large-scale atmospheric circulation

**Fig. 5:**
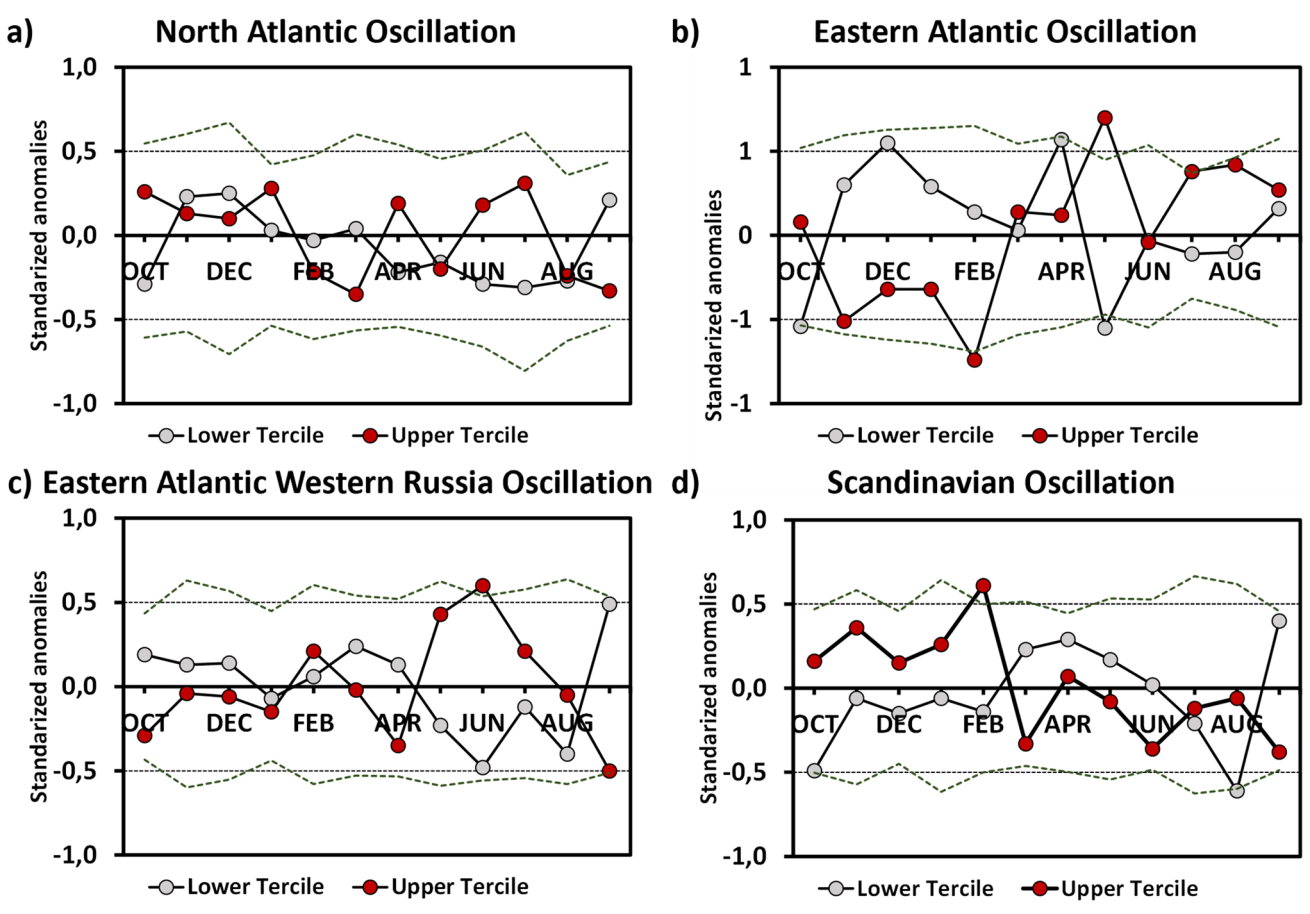
Annual cycle of monthly composites of anomalous values of the (**a**) North Atlantic Oscillation (NAO), (**b**) Eastern Atlantic Oscillation (EA), (**c**) Eastern Atlantic/Western Russia Oscillation (EA/WR) and (**d**) Scandinavian Oscillation (SCAND) with respect to low and high PC1 years. Dotted lines indicate the threshold of significant anomaly values at the 5% and 95% levels of confidence, as obtained from 10, 000 random samples

A comparative analysis was conducted on the monthly values of the main modes of low-frequency atmospheric variability in the Northern Hemisphere. The goal of this analysis was to explore potential impacts of the large-scale atmospheric circulation on wine characteristics (Marta et al., 2010; Najafi et al., 2019). It was found that May and March were pivotal months, showing climatic variability linked to the contrasting phases of the Eastern Atlantic pattern (Fig. 5).

**Fig. 6:**
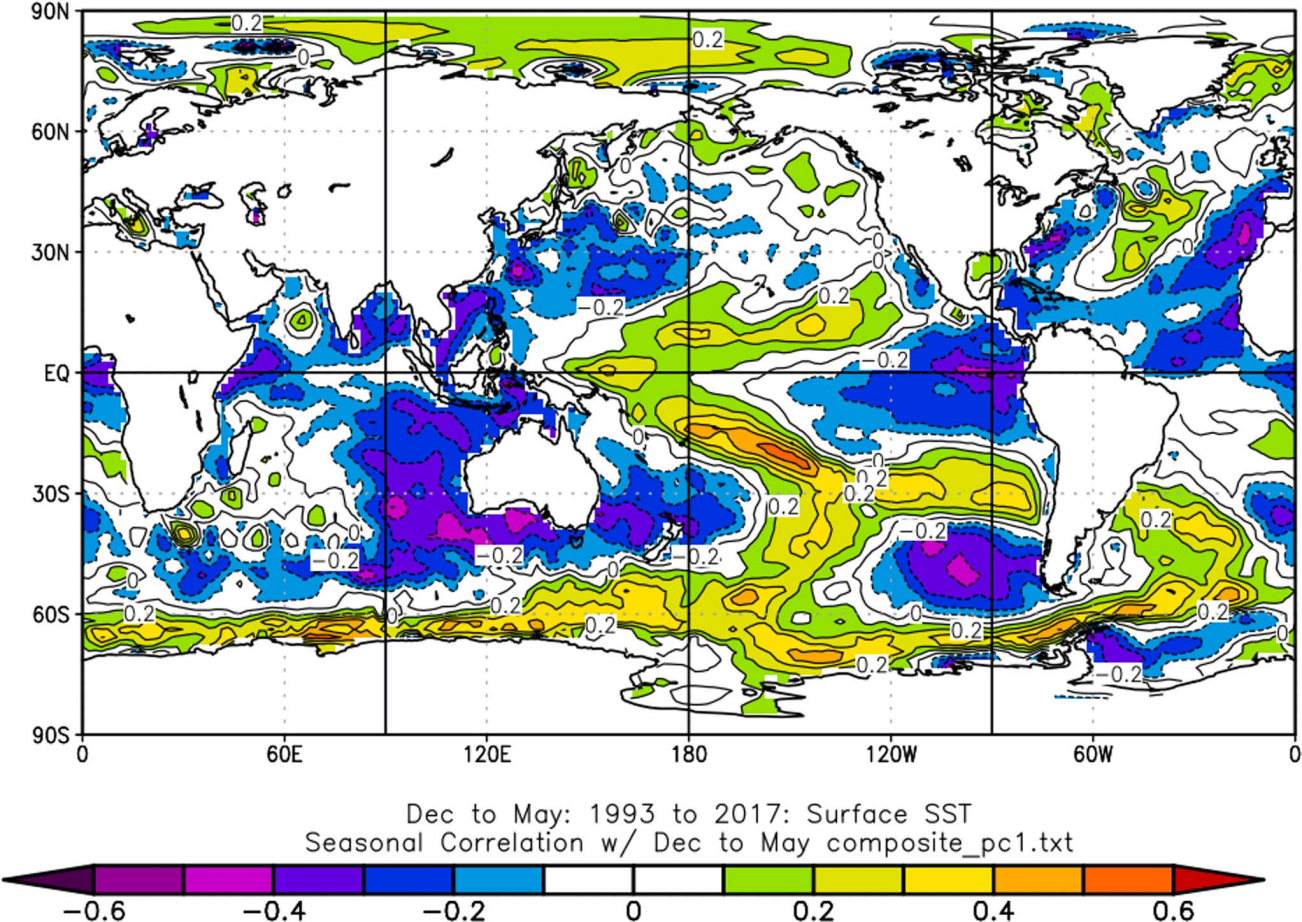
Correlation map between PC1 time series and the previous December to May Sea Surface Temperature. Image provided by the NOAA/ESRL Physical Sciences Laboratory, Boulder Colorado from their web site at http://psl.noaa.gov/. Values higher (lower) than ± 0.40 are statistically significant at the 95% significance level

To confirm the results presented by Rodó and Comín (2000), who associated the quality of five Spanish wines with the El Niño-Southern Oscillation (ENSO) through a connection with spring precipitation, the detrended time series of the three PCs were correlated with global sea surface temperature (SST) time series. Given the diversity of indices quantifying ENSO variability, SST was used, one of the most relevant components of the coupled ENSO system, as a proxy of ENSO variability. A weak negative correlation was observed between PC1 and global sea surface temperature, whose spatial pattern resembled the one of a La Niña event (Fig. 6).

## Discussion

This paper focuses on evaluating the temporal changes in various characteristics of Rioja wines and their correlation with regional climate variability. PCA was employed to derive indices representing these properties, which were then utilized to assess their association with fluctuations in regional climate variability.

Over the study period, Rioja wines displayed changes in their chemical composition and phenological cycles. These changes included an increase in alcohol content and pH, a decrease in total acidity, and earlier grape ripening, accompanied by a reduction in leaf canopy. Notably, differing trends were observed in the timing of veraison and harvest, because the timing of veraison is more dependent on natural processes, while harvest timing is also influenced by socio-economic factors.

These changes are positively correlated with the warming trend observed during the growing season across the Ebro Valley in recent decades (El Kenawy et al., 2012; Gonzalez-Hidalgo et al., 2015). Studies have shown that summer temperatures have a significant impact on the long-term changes in wine characteristics, with effects varying by region. In cooler climates, rising temperatures have been linked to higher levels sugar concentrations and alcohol, along with decreased anthocyanin concentration (which affects grape color) and acidity. This temperature increase has also been associated with shorter ripening periods and grape biological cycles (Bock et al., 2011; Meier et al., 2018). Conversely, in warmer regions, the observed temperature rise has been associated with the production of lower-quality wines (Lopez-Bustins et al., 2014; Blanco Ward et al., 2019).

The available evidence does not support the hypothesis that climate variability influences wine production and yield. The study period aligns with significant growth in the wine industry in La Rioja, driven by new management practices. These practices include the shift from rainfed viticulture to controlled irrigation, the expansion of trellised vineyards for mechanical harvesting, and changes in the regional landscape, such as increased vineyard area and plot concentration. Consequently, grape harvest volume has doubled in recent decades, accompanied by expanded storage capacity, new bottling facilities, and economic diversification in La Rioja (Lasanta et al., 2016). However, this success is not without challenges, including concerns about the loss of a centuries-old cultural landscape and a decline in grape varietals (Arnáez et al., 2012).

The impact of berry weight on grape composition and wine quality is a topic of debate within the scientific community. Wet conditions have been linked to increased berry weight and size (Williams et al., 2010). However, other factors, such as the number of berries and bunches, as well as compactness, also play a role in determining berry size (Ramos et al., 2020). The analysis suggests that berry weight behaves relatively independently of other variables. Furthermore, its correlation with local temperature and precipitation is only moderate. Significant correlations with the SPEI are observed at three, six, and twelve-month lag, but it is important to note that the SPEI index considers simultaneously average temperature and accumulated precipitation, which may diminish the importance of precipitation and weaken the relationship.

The limited correlation between precipitation levels and key compounds in Rioja wines necessitates a detailed explanation. Uriarte et al., (2015) observed that different irrigation methods in Tempranillo grape plots altered the overall acidity profile without significantly impacting pH. In contrast, Intrigliolo and Castel (2008) reported an increase in pH with irrigation. Although vineyards are tolerant to dry conditions, the answer might rest in the characteristics of spring precipitation in this area. Most precipitation occurs during convective atmospheric conditions, known for their variability in both time and space.

Efforts have been made to explore potential links between vine quality, as represented by vintages, and climate variability; however, the findings remain inconclusive, prompting further investigation. Vintage ratings serve as a common method for assessing the quality and characteristics of specific vintages through expert “blind” evaluations of appearance, aroma, and flavor. Some critics argue that these ratings may be influenced by economic factors (Johnson and Robinson 2019). Additionally, meteorological events not accounted for in this study, such as unusual temperature patterns during critical grape growth stages, can have nonlinear impacts on wine quality. For example, the delayed grape ripening in 2008, caused by a late spring, initially resulted in young Rioja wines receiving tepid reviews from experts. Nevertheless, as these wines aged, their quality improved, and they gained popularity.

The inter-annual fluctuations in LAI exhibit robust links with long-term shifts in wine composition and phenology. Elevated temperatures, conducive to the production of wines with higher alcohol content and pH levels, are inversely related to LAI values. Vineyards boasting higher LAI values tend to yield more grape bunches, while those with lower LAI values produce fewer bunches. A reduction in the number of bunches per vine leads to grapes with higher sugar content, ultimately resulting in wines with increased alcohol levels and reduced acidity. While solar radiation is essential for photosynthesis, it also increases plants’ water requirements, potentially causing water stress and issues like leaf sunburn.

May plays a pivotal role in the grape-growing cycle in La Rioja. This period aligns with the critical flowering and blooming stages of grapevines, where weather conditions oscillate between warm and dry or cool and wet, influenced by Eastern Atlantic low-frequency atmospheric variability. Stable conditions during flowering and bloom are crucial for proper flower differentiation and berry formation. Weather anomalies during the budbreak stage (March) also have a weak impact on PC1. However, warm temperatures can expedite maturation, leading to earlier budbreak due to high temperatures and dry conditions in late winter (Cunha et al., 2010). Finally, the period surrounding veraison is critical for fruit set, necessitating dry and warm conditions, ample sunlight, and minimal temperature fluctuations to enhance sugar concentration (Nemani et al., 2001). These optimal conditions are typically met during the summer months in La Rioja.

Furthermore, the data revealed a subtle yet noticeable correlation between the detrended values of the first principal component (PC1) and an SST pattern that closely resembles El Niño–Southern Oscillation (ENSO) dynamics. Specifically, high values of PC1 correspond to cold SST anomalies, while low values align with warm SST anomalies in the eastern equatorial Pacific. This suggests that the negative phase of ENSO, known as “La Niña,” may lead to warmer and drier conditions during the spring and summer months in La Rioja. Conversely, the opposite may occur during the “El Niño” phase. While some studies have linked winter precipitation to ENSO (Sordo et al., 2008), it is generally agreed that the impact of “La Niña” on the Iberian Peninsula is more pronounced in spring. For instance, Rodó et al., (1997) identified positive correlations in the continental areas of eastern Spain, with this relationship strengthening towards the end of the 20th century. Vicente-Serrano (2005) and Lorenzo et al., (2011) noted significant correlations between drought conditions and “La Niña” years. The influence of ENSO on regional temperatures remains uncertain; however, Frías (2010) suggests a possible link between La Niña and the occurrence of summer heatwaves in southern Spain. This paper does not aim to explain the mechanisms by which La Niña’s influence extends to mid-latitudes. Nevertheless, a northward shift of the jet stream can lead to the development of a region with higher geopotential heights and enhanced anticyclones during the following summer, particularly in the context of a La Niña event. This phenomenon has been associated with an increased risk of extreme heat events in mid-latitudes (Luo and Lau 2020).

The findings of the study are poised to advance the comprehension of the enduring effects of global warming and assist in forecasting the necessary actions to sustain the economic viability of Rioja wines at their current standards. Recent climate patterns in La Rioja correspond with the anticipated climate scenarios for the Iberian Peninsula, indicating a notable decline in the region’s suitability for viticulture, particularly in northwest Spain (Lorenzo et al., 2016; Gaitán and Pino-Otín 2023; Ramos and Yuste 2023). Additionally, Ramos and Martinez de Toda (2020, 2022a, 2022b) have predicted that Rioja wines will encounter more acute water shortages, an advancement in phenological stages by up to 15 days, and modifications in grape composition.

It is essential to recognize the constraints of this analysis. One significant limitation is the omission of meteorological occurrences such as hailstorms, which can substantially impact crops. In a generally warmer climate, the frequency and intensity of such events are likely to escalate (Gascón et al., 2015). Further investigation is warranted to comprehend the impact of climate change on vineyard health, particularly concerning pest cycles. With the region shifting towards a warmer southeast climate, there is a noticeable uptick in the frequency of pest cycles (personal communication, D. Martín Martínez, Spanish Ministry of Agriculture, Fishing, and Food).

Moreover, the Rioja wine industry must adjust to evolving consumer preferences. The emergence of new social trends and the surging popularity of low-alcohol beverages have led to a surge in the consumption of “light” wines. Wines with higher alcohol content often boast a more robust flavor profile, which may not resonate with a significant portion of contemporary consumers (Bucher et al., 2018, 2019). Furthermore, high-alcohol wines are at times perceived unfavorably due to potential health repercussions (Anderson et al., 2021) and may encounter additional taxes in certain nations. These shifting consumer inclinations could result in the exclusion of high-alcohol wines like Rioja from the global market, potentially diminishing the profitability of vineyards in the region.

## Conclusions

The aim of this research is to investigate the correlation between wine production, quality, chemical composition, and phenological development of wines from the DOCa Rioja region considering recent climate patterns spanning from 1993 to 2017. The increasing temperatures experienced during the growing season have resulted in wines exhibiting elevated alcohol levels and pH values, reduced acidity, and notable phenological shifts, leading to earlier grape maturation and diminished foliage density. Of particular significance is the month of May, which plays a pivotal role due to its association with low-frequency atmospheric variability originating from the Eastern Atlantic, exerting a substantial impact on the success of the harvest. Additionally, this study delves into potential connections with fluctuations in tropical climates. These discoveries offer valuable insights for crafting tailored strategies for vineyard management practices, especially for wines characterized by higher alcohol content that may encounter challenges in global markets.

## Data Availability

Climatology and remote sensing data will be made available upon reasonable request. However, the wine data used in this study are confidential and cannot be shared publicly.
